# Molecular Dynamics Simulations of Oil Detachment from Hydrophobic Surfaces by Using Janus Nanoparticles

**DOI:** 10.1021/acs.jpcb.5c01850

**Published:** 2025-06-23

**Authors:** Tomasz Staszewski, Małgorzata Borówko

**Affiliations:** Department of Theoretical Chemistry, Institute of Chemical Sciences, Faculty of Chemistry, 49686 Maria Curie-Skłodowska University in Lublin, Lubin 20-031, Poland

## Abstract

Janus particles composed of two parts with different chemical properties can be used for enhanced oil recovery. We investigated the role of Janus dimers in the process of detachment of oil aggregates from hydrophobic solid surfaces using molecular dynamics. Large-scale simulations were performed for different sets of parameters characterizing the system. The effects of interactions between Janus particles and a solid surface and with an oil droplet were considered. We found two main mechanisms of enhanced oil removal: the “kidnaping” of oil aggregates by Janus nanoparticles from the substrate and the competitive adsorption of nanoparticles at the solid surface. In the case of weak affinity of Janus particles with the substrate, the first mechanism dominated, whereas when the affinity was strong, the second mechanism played an important role. We showed how the amphiphilicity of Janus particles and their concentration influence the shape and internal structure of oil aggregates adsorbed on a hydrophobic surface. The high amphiphilicity of Janus particles and their increased concentration promote the process of removing oil from the surface. We analyzed the time evolution of the system after the addition of Janus particles in detail. In the “kidnaping” process, the flat oil aggregate left the surface as a large, sandwich-like aggregate, while the adsorption of Janus particles on the surface caused it to break up into small pieces that left the surface as nearly spherical droplets.

## Graphical abstract

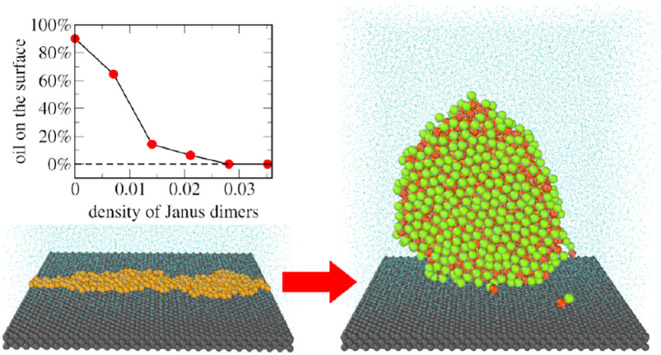

## 1 Introduction

The economy’s demand for oil is constantly increasing due to population growth and industrial development. Therefore, it is necessary to research new methods of increasing oil production in an economically viable way. For this purpose, various methods for enhanced oil recovery (EOR) have been developed.^1,2^ All of these techniques have both advantages and limitations. The chemical-based methods seem to be especially promising. During chemical flooding, various additives are injected into the reservoir, including polymers, surface active agents, nanoparticles, and their mixtures. However, polymers have low injectability and change their properties (viscosity) in the reservoir, while surfactants are strongly adsorbed on the rock surface, which results in their high consumption and increased costs. In recent years, a new class of active substances, namely, various nanoparticles (NPs), has attracted the attention of researchers.^1–6^ Nanoparticles, due to their unique physical and chemical properties, can be successfully used in EOR. The surface-to-volume ratio of nanoparticles is very high due to their very small size, and they can enter the pores without being trapped. In comparison to polymers and surfactants, NPs have higher charge density, higher surface area, lower adsorption on the rock surface, and lower scaling formation. Due to their small size, they do not plug pores. Moreover, NPs are more environmentally friendly in comparison to other chemicals used in EOR. However, some studies have reported the low efficiency of simple NPs, so their mixtures with polymers or surfactants are used.

An innovative solution is the use of Janus nanoparticles (JNPs) which consist of two parts with different chemical properties.^3^ They combine the advantages of surfactants and NPs. The behavior of Janus nanoparticles at liquid/liquid interfaces and their technological applications have been discussed in several reviews.^7–9^

Numerous studies have confirmed that JNPs have a huge impact on improving oil production by (i) interfacial tension reduction, (ii) water viscosity increment, (iii) substrate surface wettability alteration, and (iv) oil displacement mechanisms.^1^

In the water flooding process, the efficiency of oil recovery is low due to the high interfacial tension at the oil/water phase boundary and also the low value of water viscosity. Under such conditions, the capillary pressure traps oil droplets in the porous rock. However, the addition of Janus particles reduces the interface tension between the water and oil phases, leading to the release of oil droplets from the pores. The amount of remaining oil in the reservoir decreases by increasing the capillary number defined as *N*_c_ = *ν*μ/γ, where *ν* is the velocity, μ is the fluid viscosity, and γ is the interfacial tension at the oil/water interface. Thus, an increase in the viscosity of the eluting fluid or a decrease in the interfacial tension can result in improved oil recovery. Moreover, stable oil displacement occurs when the oil is more mobile than the water phase. For this purpose, the water mobility should be reduced through an increase in water phase viscosity. Recent studies have shown that JNPs can increase the viscosity of aqueous solutions.^10^ Reducing the residual oil saturation can also be achieved by changing the wettability of the surface from oil-wet to water-wet. Then, the oil droplets could detach from the surface. Janus particles can strongly alter the surface wettability due to their high surface energy resulting from a large surface-to-volume ratio and amphiphilicity. The oil recovery mechanism is as follows. Janus particles adsorb at the oil/water interface, self-assemble, and cover the droplet, which results in its detachment from the substrate. As the Janus particles reduce the interfacial tension at the oil/water interface, a Pickering emulsion is formed.

Other oil removal mechanisms were proposed by Luo et al.^11^ who studied the use of graphene-based Janus nanosheets in this process. They noticed that the nanoparticles were adsorbed on the rock surface and formed a wedge-shaped layer in the oil–water–solid contact area. The wedge film generated a structural disjoining pressure. When the disjoining pressure was stronger than the oil droplet, adhesion oil was removed from the substrate. They suggested two oil displacement mechanisms for Janus nanosheets: (i) climbing film growth mechanism and (ii) slug-like displacement mechanism.^11^ In the first case, Janus nanoparticles formed a film at the oil-rock interface, which grew gradually over time. The adsorption of Janus nanoparticles caused the formation of the three-phase zone (nanoparticles, oil, and rock surface) and IFT reduction. The latter, in turn, led to Marangoni stress in this three-phase zone. The film gradually grew due to the ongoing supply of nanoparticles from the fluid and oil encapsulated on the rock surface. Finally, the induced Marangoni stress moved the oil forward, detaching the oil from the rock surface. Slug-like displacement mechanism occurred as a result of forming an elastic interfacial film between the oil and water phases. Under strong hydrodynamic conditions, the formation of a solid-like interfacial film led to the separation of two phases. This film had considerable bending resistance. Janus particles accumulated at the interface and formed a film that gradually climbed the wall, encapsulated the oil phase, and pushed the oil in the flow.^1^ The physicochemical properties of carboxyl/alkyl composite silica-based amphiphilic Janus nanosheets were systematically investigated.^12^ It was found that the formation of a climbing film and a high-strength elastic oil–water interface by these nanocomposites may play an important role in the EOR mechanism. Quite recently, Shi et al.^13^ proposed the innovative utilization of Janus SiO_2_ particles amalgamated with graphene oxide for the extraction of residual hydrocarbons from increasingly depleted reservoirs. Their core flooding experiments corroborated the superior efficiency of the nanocomposites in augmenting oil recovery compared with traditional methodologies, which was due to the improvement of both emulsifying ability and amphiphilicity.

Although experimental studies have shown the most important macroscopic features of systems used in the EOR, they do not provide a complete microscopic picture of this process. The molecular level is not usually accessible by experimental equipment. Computer simulations emerge in this scenario as an alternative to access information at an atomic level and for the rational design of nanomaterials with specific properties for the EOR. Molecular simulations were widely used to study the mechanisms underlying the EOR and the potential role of nanoparticles in this process. Most of these simulations involved spherical particles with a chemically homogeneous surface.^14–19^ Liu et al.^14^ investigated the mechanism of oil detachment from silica surfaces in aqueous surfactant solutions. They showed that this process could be divided into three stages: (i) the shrinking of the contact line due to the decrease of interfacial tension caused by the adsorption of surfactant at the oil–water interface; (ii) separation of oil from the substrate near the contact line by water diffusion; and (iii) a decrease of contact radius and droplet detachment. The contact line motion was caused by an imbalance of the interfacial tensions at the three-phase contact line. However, this oil detachment could not be simply explained by the above scheme because the imbalanced interfacial tension force would point in opposite directions. In this detachment mechanism, the formation of water molecular channels was a crucial step.^14^ In this way, water molecules could penetrate the oil–water interface. The propagation of water molecules on the silica substrate accelerated the removal of oil molecules from the substrate. Wang and Wu^15^ studied oil droplet detachment from solid surfaces immersed in charged nanoparticle suspensions. Their results demonstrated that the charge of the nanoparticles played a significant role in this process. They found that when the charge on each particle exceeded a threshold value, the complete detachment of the oil droplet occurred spontaneously. Tang et al.,^16^ used molecular dynamic simulations to investigate the surfactant flooding-driven oil detachment in nanosilica channels. In the next paper,^17^ the oil detachment from the hydroxylated silica surface was considered. The focus was paid on: the effects of surfactants, electrostatic interactions, and water flows on the water molecular channel formation. Moreover, the synergistic effects of surfactants and nanoparticles on oil–water interfacial behaviors were studied using molecular simulations.^17–20^ Liu et al.^20^ studied the mechanism of oil detachment from surfaces of different hydrophobicity and found that the oil detachment process became slower and the oil-solid contact angle was smaller with the increasing hydrophobicity of the surface.

Relatively fewer simulations were performed for oil droplet detachment by nanoparticles with modified surfaces.^21–24^ Liang et al.^21^ compared the oil displacement properties of nanoparticles with grafted hydrophobic, hydrophilic, and a combination of hydrophilic and hydrophobic chains. They showed that the nanoparticles with mixed hydrophilic and hydrophobic chains had an optimal EOR capability. The effects of salt and nanoparticles on oil–brine interfacial properties were also investigated for hydrophilic, hydrophobic, and Janus nanoparticles.^22^ However, Chang’s group^23^ explored the displacement mechanism of trapped oil in a rough channel by injecting different nanofluids. They proved that hydrophilic and Janus nanoparticles enhanced the oil displacement efficiency by different mechanisms. In subsequent papers,^25,26^ the dynamic elution of oil from nanopockets using Janus nanoparticles was analyzed. The crucial stages in oil recovery by Janus nanoparticles, termed the “adsorption invasion process”, were distinguished, which comprised anchoring onto the surface, pinning at the edge, and entering into the pocket. They focused, however, on the effects of the surface. Zhou et al.^24^ studied the influence of modified SiO_2_ nanoparticle, with an increasing number of polar groups on their surfaces, on the properties of the oil–water interface and the detachment of oil droplets from an oil-wet surface. They confirmed the formation of water channels, which supported the detachment of the oil droplets. Their analysis also indicated that in terms of oil displacement efficiency, the thickness of the interfacial layer had a more significant impact than interfacial tension reduction.

The research on enhanced oil recovery was supplemented by numerous molecular dynamics studies on the behavior of nanoparticles at the liquid–liquid interfaces^7–9,27,28^ and the wettability of solid surfaces.^29–35^

Despite significant progress in research on the use of JNPs to enhance oil recovery, many specific issues require investigation. Possible mechanisms for recovering oil from rock are still under debate. To obtain a deep understanding of the behavior of JNPs in the EOR process, comprehensive simulation studies of the relevant model systems are necessary. An essential issue is to explain the retention of oil on the surfaces of solids under various conditions. It requires very time-consuming simulations. In the case of fully atomistic simulations,^18,20,21,23,24^ this aspect is particularly important. The simulations performed so far concerned specific systems involving solids, oil droplets, and nanofluids. In particular, the behavior of rather randomly selected nanoparticles in such systems was studied. We noticed some gaps in this research. First, in the majority of investigated particles, different grafted chains (or functional groups) were distributed randomly on their surfaces. Typical Janus particles with two regions of different properties on the surface have rarely been considered.^22,23,35^ Second, only the spherical nanoparticles were studied as active agents. However, it is well known that the shape of the nanoparticles strongly influences their behavior on the liquid/liquid interface. Janus particles can adopt different shapes, such as snowmen, dumbbells, dimers, hybridized-like orbitals, and mushrooms, with clear geometrical and topological asymmetries. Unique properties of the snowman-type Janus particles were discussed in the review by Honciuc.^36^ Furthermore, no systematic studies have been performed on the influence of different interactions on the behavior of oil droplets on solid surfaces as well as on the influence of Janus particle concentration on droplet detachment. In our research, we tried to fill these gaps.

In this paper, we present the results of large-scale molecular dynamics simulations based on a simple coarse-grained model of the system consisting of oil/solvent suspension of Janus dimers^28,36,37^ and the solid surface. The application of Janus dimers in oil recovery has not yet been studied so far.

We performed simulations of the adsorption of oil droplets on various surfaces, from hydrophobic to hydrophilic. The obtained results were qualitatively consistent with previous simulations^20,21,31,33–35^ and experiments.^5,33,34,38^ This confirmed the predictive ability of our model. Next, we concentrated on the mechanisms of detachment of flat oil aggregates from the strongly hydrophobic substrate under the influence of the addition of Janus dimers. Such a detachment is difficult in a static system without fluid flow. We considered two mechanisms of how Janus dimers affected interfacial performance and oil displacement: (i) “kidnaping” of oil aggregates from the surface by Janus dimers and (ii) competitive adsorption of Janus dimers on the substrate.

The “kidnaping” process contained two stages: the desorption of oil aggregates from the substrate and the formation of oil droplets in the solvent (emulsification). This mechanism has already been reported for hairy nanoparticles.^21,24^ However, we studied systematically how a change in energy of interactions between Janus dimers and oil molecules affected the structure of the system, the location of the oil aggregate, and the dynamics of the oil detachment. We proved that an increase of Janus dimer amphiphilicity considerably enhanced oil detachment. We found that there was a threshold value of amphiphilicity at which Janus dimers can draw a droplet into the aqueous phase. We observed the formation of unique sandwich-like structures by oil aggregate and Janus dimers in the detachment process.

The second driving force for oil removal from the substrate was the adsorption of Janus nanoparticles on the solid surface. This effect was omitted in most of the previous simulations. In this study, we showed the influence of the concentration of Janus dimers on the behavior of oil aggregates on the surface. Moreover, we analyzed the successive stages of the detachment of the oil cluster in detail. In this case, the flat oil aggregate changed shape on the surface and left it in the form of almost spherical droplets.

## 2 Model and Methodology

### 2.1 Model

We studied a static system composed of polymers (*O*) and polar solvent molecules (*W*) in contact with the flat surface of a solid. We introduced such thermodynamic conditions (density and temperature) that the polymer (oil) forms droplets dispersed in a solvent. To this system, we inserted Janus nanoparticles. A simple coarse-grained model was used to mimic all species. Polymer segments and solvent molecules had the same diameters, σ. Polymer chains were built of *M* segments. Janus particles were dimers composed of two spheres (segments) *A* and *B* that had identical sizes (diameters σ_*A*_ = σ_*B*_) and different chemical properties. The solid was a crystalline lattice fcc consisting of “atoms” (*S*) of diameters σ_*S*_ = σ. The crystalline plane (001) corresponds to an exposed surface.

The chain connectivity was ensured by a finitely extensible nonlinear elastic (FENE) segment–segment potential

1 $$U_{FENE}=-\frac{k}{2}R_{0}^{2}\ln\left[1-{\left(\frac{r}{R_{0}}\right)}^{2}\right]$$

where *r* denotes the separation distance between the segments, *k* is the spring constant, and *R*_0_ is the maximum possible length of the spring. The standard parameters of the binding potential (1) were assumed: *k* = 30 and *R*_0_ = 1.5.

The interactions between all “atoms” (solvent molecules, chain segments, and solid molecules) were modeled via the shifted-force Lennard-Jones potential^39^

2 $$u=\{\begin{array}{l} 4\varepsilon_{\mathrm{ij}}\left[{\left(\sigma_{\mathrm{ij}}/r\right)}^{12}-{\left(\sigma_{\mathrm{ij}}/r\right)}^{6}\right]-\Delta u\left(r\right),r<r_{\mathrm{cut}\left(\mathrm{ij}\right)}\\ 0,\mathrm{otherwise} \end{array}$$

where

3 $$\Delta u\left(r\right)=u\left(r_{\mathrm{cut}\left(\mathrm{ij}\right)}\right)+\left(r-r_{\mathrm{cut}\left(\mathrm{ij}\right)}\right)\partial u\left(r_{\mathrm{cut}\left(\mathrm{ij}\right)}\right)/\partial r$$

and *r*_cut(*ij*)_ is the cutoff distance, σ_*ij*_ = 0.5­(σ_*i*_ + σ_*j*_), (*i*, *j* = *O*, *W*, *A*, *B*, *S*), *ε*_*ij*_ denotes the parameter that characterized interaction strengths between spherical species *i* and *j*. The indices *O*, *W*, *A*, *B*, and *S* corresponded to oil, solvent, segments of Janus dimer, and solid, respectively. As in the previous works,^9,35^ the cutoff distance was used to switch on or switch off attractive interactions. For attractive interactions, *r*_cut(*ij*)_ = 2.5σ_*ij*_, while, for repulsive interactions, *r*_cut(*ij*)_ = σ_*ij*_. The gravity effect was assumed to be negligible.

We used the standard units commonly defined in coarse-grained simulations.^27–29,35,40^ The diameter of solvent atoms was the distance unit, σ, the mass of a solvent atom was the mass unit, *m*_*W*_ = *m*, while the solvent–solvent energy parameter, ε_*WW*_, was the energy unit. The unit of time was τ = σ­(*m*/ε)^1/2^.

We also introduced the reduced dimensionless quantities:^30,35,40,41^ reduced distances l* = *l*/σ, reduced energies E* = *E*/ε, reduced time t* = *t*/τ, and the reduced temperature T* = *k*_B_ *T*/ε, where *k*_B_ is the Boltzmann constant.

Moreover, we used the reduced density of the *k*th component defined as ρ_k_^*^ = ρ_*k*_σ_*k*_^3^, where ρ = *N*_*k*_/*V*, was its number density, *N*_*k*_ is a total number of “atoms” *k*, and *V* is the volume of the system, as well as the average reduced density of Janus dimers ρ_AB_^*^ = ρ_A_^*^ + ρ_B_^*^.

The coarse-grained models are very flexible and can be easily adapted to different real-world systems. For example, we can assume that the solvent segment (*W*) corresponds to one or two water molecules, while the oil segment contains a few CH_2_ groups.^42^ The Lennard-Jones energy parameter can be modified by a dimensionless coefficient to create a wide spectrum of interactions.^31^ In this way, it is possible to take into account the brine effect or generate different surfaces, from hydrophilic to hydrophobic.

Here, we used parameters similar to those applied in previous coarse-grained simulations of the interfacial systems involving nanoparticles and polymers.^27,35,41,42^ The model validation consisted of a thorough analysis of the dependencies between the values of our parameters and their comparison with those used in molecular dynamics^27,35,41^ and DPD simulations.^42^ The parameters used in our simulations are described in the next subsection.

Our model does not represent a specific chemical system, but rather a simple approach widely used in the literature to easily tune the behavior of the interface.^40^

Based on a model that ignores details of particle structure, one can relatively quickly study the behavior of complex systems for many sets of parameters. Moreover, the use of a coarse-grained model allows for the comparison of simulation results with predictions of various theoretical approaches, including classical functional density theory.^37^

### 2.2 Simulation Protocol

The LAMMPS package^43,44^ was employed for molecular dynamics (MD) simulations. The temperature was kept constant at T* = 1 by the Nose-Hoover thermostat. The equations of motion were integrated by using the velocity Verlet algorithm. The time step was set to Δt* = 0.002. Equilibrium was monitored by analyzing the energy as a function of time. Each system was equilibrated for at least 10^8^ time steps until its total energy reached a constant level. The production runs had at least 10^7^ time steps.

Simulations were carried out in a rectangular box of reduced dimensions equal to *L*_x_^*^ = *L*_y_^*^ = 40 and *L*_z_^*^ = 28 or *L*_z_^*^ = 43 along axes *x*, *y*, and *z*, respectively. In the case of a bulk system, standard periodic boundary conditions in all directions were assumed. For the adsorption systems, however, the periodic conditions were applied only in the *x* and y directions. The box was closed at the top by a reflective wall. Three layers of “atoms” *S* that formed the fcc crystalline lattice were located at the bottom of the box to mimic the solid phase.

Simulations were performed for different sets of energy parameters. We considered that segments “*i*” and “*j*” were inert to each other if the interactions between them were purely repulsive. In this case, we assumed ε_ij_^*^ = 1 and *r*_cut(*ij*)_ = σ_*ij*_.

W carried out simulations for: (i) the bulk mixture oil-solvent, (ii) the adsorption of oil droplets from the oil-solvent suspension on different solid surfaces, and (iii) the detachment of oil aggregates from the strongly hydrophobic surface.

We started with the simulation of the bulk system consisting of solvent molecules (*W*) and decan molecules (*M* = 10) that mimicked oil (*O*). The reduced density of the solvent was ρ_W_^*^ = 0.7. We assumed that self-interactions between fluids (oil–oil and solvent–solvent) were attractive with ε_WW_^*^ = 1.0, ε_OO_^*^ = 1.5, while the cross-interactions were repulsive.^27,28^ The density of polymer molecules (oil) was very low in comparison to the density of solvent molecules (solvent-rich mixtures). Under the considered conditions (temperature and density), in the bulk oil-solvent mixtures, phase separation occurred the phase separation. The oil molecules formed almost spherical droplets that were dispersed in the solvent.

To simulate the adsorption of the oil droplets near different surfaces, such “bulk” configurations were used as the initial configurations. In the previous works,^29–31^ the simulations started from “artificial droplets” located on the substrate (e.g., cubic aggregates). Our approach seemed more realistic.

All simulations were carried out for strong attractive oil-substrate interactions; we put ε_OS_^*^ = 1.5 and changed water-surface interactions (ε_WS_^*^).

In the third series of simulations, we focused on the strongly hydrophobic surface with repulsive *WS* interactions.

Removing an oil stain from such a surface is very difficult. For this purpose, we added Janus dimers to the system. We considered small Janus particles, similar to short surfactant molecules.^36,45^ The reduced diameters of the segments of Janus dimers were set as σ_A_^*^ = σ_B_^*^ = 1.5. The behavior of similar Janus dimers at the oil/water interface was discussed in our previous papers.^9,28,37^ We studied the behavior of systems involving Janus dimers inert with respect to the substrate or those inert with respect to oil.

We carried out simulations for systems containing different numbers of Janus dimers, *N*_*AB*_ = 100, 200, 300, 400, 500, 600, 800. In this case, we started from the equilibrium configuration with an oil droplet attached to the substrate, and we inserted Janus particles into such a system from above and observed its evolution over time until a new equilibrium was reached.

Each Janus dimer consisted of two different segments, *A* and *B*. This meant that the number of energy parameters that determine the system equilibrium considerably increased. To limit the number of these parameters, we assumed that the same segments attracted each other (ε_AA_^*^ = ε_BB_^*^ = 1.5), but *AB* interactions were repulsive.^28^ We assumed that segments *A* attracted solvent molecules (ε_AW_^*^ = 1.0), and they were inert with respect to the oil chains.

The OVITO 3.0.0 software^46^ was used for visualization.

## 3 Results and Discussion

### 3.1 Adsorption of Oil Droplets on Solid Surfaces

In this section, we show that our simple coarse-grained model reflects well the behavior of oil droplets adsorbed at solid surfaces. In addition, we discuss here the observables that subsequently serve to characterize systems involving Janus particles.

The adsorption of oil aggregates on the silica surface in the water phase results from the interplay between all interactions in the systems These interactions determine values of macroscopic quantities characterizing droplets, such as surface tensions of three phases, adhesion work, the contact angles, the line tension, and the length of the three-phase contact line. The thermodynamic fundamentals of the wettability of solids were recently discussed in the review by Małecka et al.^38^ However, the competition between oil-surface and water-surface interactions plays a dominant role.^35^ In the framework of our model, the behavior of oil droplets can be modeled by changing the Lennard-Jones parameters ε_OS_^*^ and ε_WS_^*^. As has already been mentioned, we assumed that oil molecules were strongly attracted by the substrate (ε_OS_^*^ = 1.5) and gradually changed the affinity of the surface with respect to water: from hydrophobic to hydrophilic.

In Figure 1, we present equilibrium configurations of the system for different values of ε_WS_^*^. On a considerably hydrophobic surface (a), the oil droplet adsorbs as an almost flat aggregate. We see here that the oil stain spilled on the surface. As water-surface interactions strengthen (as ε_WS_^*^ increases), the shape of the adsorbed oil aggregates changes very clearly. The oil droplet adsorbs on the surface in the form of compact multilayer structures. At considerably hydrophilic surfaces (c, d), after adsorption, the droplet almost retains its original spherical shape; the surface aggregate resembles a truncated and flattened ball. However, for strongly hydrophilic surfaces, the oil droplet does not adsorb at all (e). The contact angle increases from 0° (a) to 180° (d), which corresponds to a change in the surface wettability, from oil-wet to water-wet.

**Figure 1:**
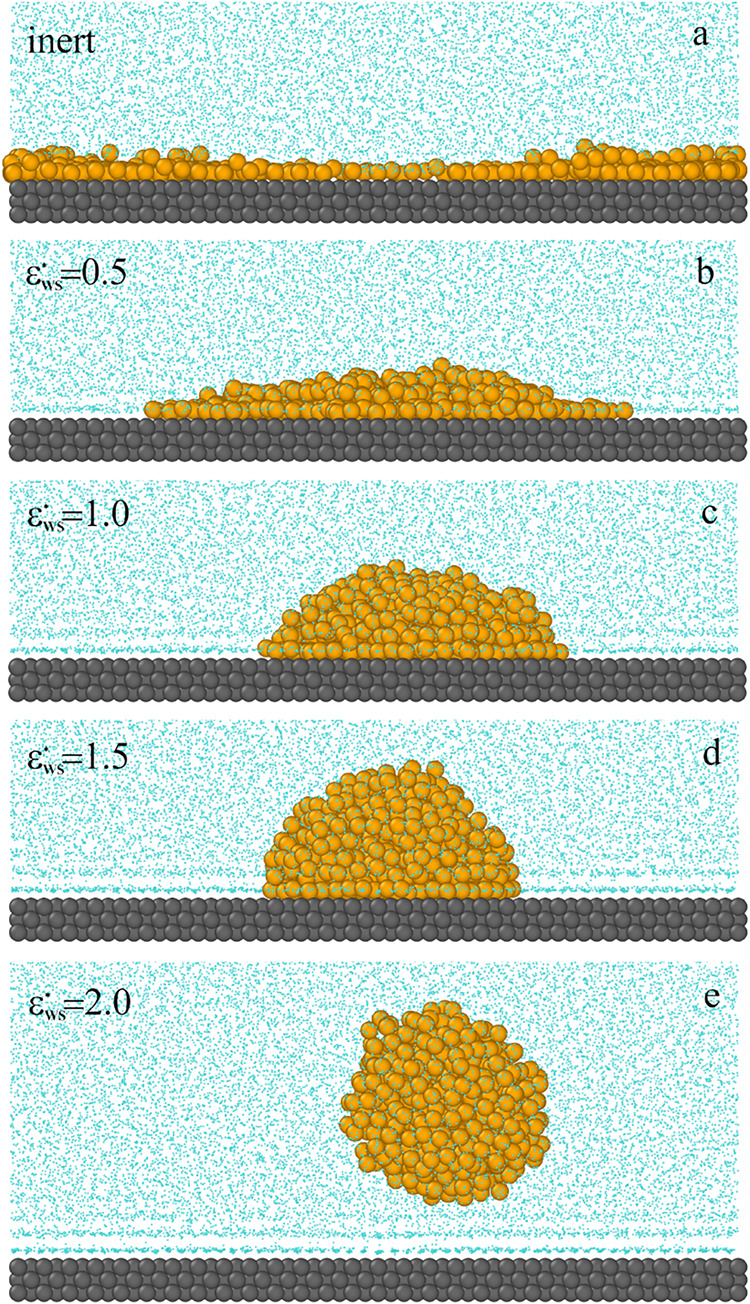
Examples of the equilibrium configurations of oil aggregates adsorbed on the surfaces of different strengths of aqueous solvent–substrate interactions—(a) the inert (hydrophobic) surface, and hydrophilic substrates with ε_WS_^*^: 0.5 (b), 1.0 (c), 1.5 (d), 2.0 (e). The yellow spheres correspond to the oil segments (*O*), the light blue spheres represent the solvent (*W*), and the gray spheres are for the solid. For greater clarity of the drawing, the solvent molecules have been reduced.

In our previous paper,^35^ we modeled the shape of adsorbed droplets changing the parameter ε_OS_^*^ and obtained the analogous results. Then, we performed a detailed analysis of the structure of adsorbed oil droplets using shape metrics. Here we will limit ourselves to showing the density profiles for different surfaces.

In Figure 2, the local density profiles of polymer segments (a) and solvent molecules (b) along the *z*-direction are plotted. To show all even very subtle features of the density profiles, the density axes are scaled logarithmically. Let us analyze the structure of adsorbed oil aggregates in detail. For the strongly hydrophobic surface, the profile (black line) has only two well-pronounced maxima: the very high peak at z* = 2.50 and the much lower one at z* = 3.36. In this case, the oil segments form two adsorption layers. When ε_WS_^*^ increases, successive peaks appear in the profiles, reflecting the formation of thicker surface aggregates, then the segment density continuously decreases to zero. Changes in the density of the oil are accompanied by corresponding changes in the density of the solvent, shown in Figure 2b. The solvent profiles are typical for a liquid, we see here relatively sharp peaks of decreasing heights. Comparison of oil density profiles (a) and solvent density profiles (b) confirms the competitive character of adsorption. An increase in ε_WS_^*^ causes a decrease in oil density but an increase in solvent density near the substrate.

**Figure 2:**
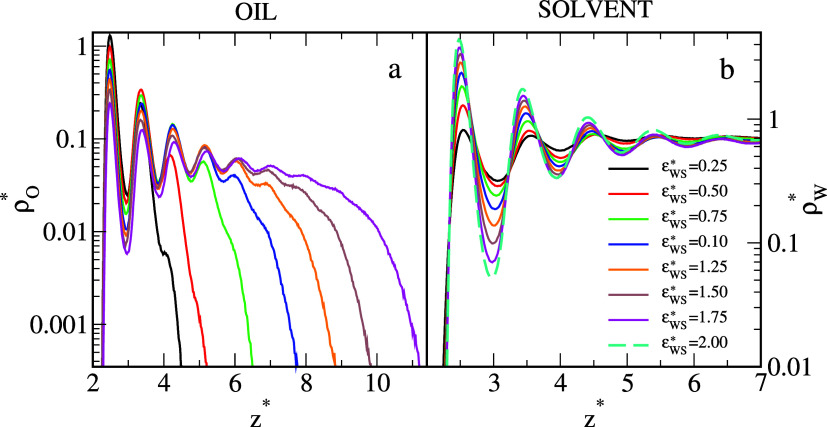
Local density profiles of the oil segments (a) and solvent molecules (b) on the surfaces with different strengths of solvent–substrate interaction—inert surface (black), and hydrophilic substrates with ε_WS_^*^: 0.25 (red), 0.5 (green), 0.75 (blue), 1.0 (orange), 1.25 (orange), 1.5 (brown), and 1.75 (magenta). In (b), the solvent density profile is also plotted for ε_WS_^*^ = 2.0 (dashed turquoise line). In this case, the oil droplet does not attach to the surface. The abscissas are scaled logarithmically.

Interestingly, for the most hydrophobic surface, the successive peaks in the density profile of water have almost the same heights. More interspaces between oil segments are exposed to water, so it might be easier to form a “water channel” in the oil aggregate adsorbed at a strongly hydrophobic surface. Similar results have been reported for silica surfaces modified with different alkanes.^20^

To characterize the behavior of oil chains adsorbed on the surface, we also calculated the average z*-coordinate of the center of mass of oil segments (*z*_com_^*^) and the number of oil segments that “touch” the surface (*N*_*O*_^(*S*)^). The latter denoted the number of oil segments that are located at a distance from the surface smaller than 0.7. We present the results in a normalized form as the fraction of the number of segments in contact with the substrate *f* = *N*_*O*_^(*S*)^/*N*_*O*_, where *N*_*O*_ was the total number of oil segments.

Figure 3 shows that as the water-surface interactions become stronger, the average z*-coordinate of the center of mass of oil segments increases and, inversely, the number of oil-surface contacts decreases. For ε_WS_^*^ = 2, the number of oil-surface contacts reaches zero, while *z*_com_^*^ increases abruptly because the droplets do not adsorb on this surface (e). It should be noted that an increase in the average coordinate z* of the center of mass of the oil segments is equivalent to a rise in the contact angle of the oil aggregate (compare Figures 1 and 3).

**Figure 3:**
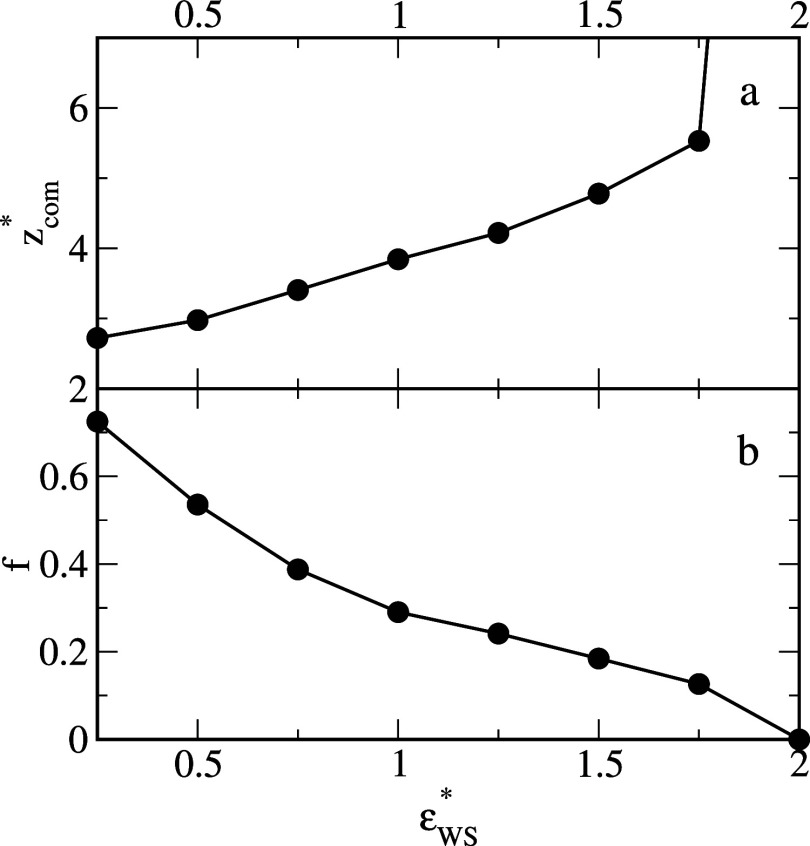
Average *z*-coordinate of the center of mass of oil segments, *z*_com_^*^, (a) and the fraction of the number of oil segments which “touch” the surface, *f*, (b) as functions of the energy parameter characterizing the solvent–substrate interactions, ε_WS_^*^.

Our simple coarse-grained model reflects the behavior of oil droplets on solid substrates. These results are qualitatively consistent with earlier simulation^15,20,21,32–35^ and experimental observations.^5,33,34,38^

### 3.2 “Kidnaping” of Oil Aggregates from the Substrate by Janus Nanoparticles

We discuss here the results for a strongly hydrophobic surface (repulsive *WS* interactions). The mechanism of oil removal from such substrates still remains unclear.^20,24^ In this case, the oil droplet spreads on the surface and forms a flat oil aggregate (compare Figure 1a). To such a system we inserted Janus dimers and observed the transformation of the oil cluster.

In this series of simulations, we assumed that both segments of Janus dimer were inert with respect to the substrate, and *BW* interactions were repulsive.

We begin with a discussion of the effects of Janus nanoparticle-oil interactions. For this purpose, we investigated the systems with the fixed concentration of Janus dimers, ρ_AB_^*^ = 0.042, and changed the affinity of Janus particles to the oil molecules. This affinity was characterized by the parameter ε_BO_^*^. We assumed that the other pair interactions remained unchanged. As mentioned, the segments *A* attracted solvent molecules (ε_AW_^*^ = 1.0) and they were inert with respect to the oil chains.

Unique interfacial properties of Janus nanoparticles result from their amphiphilicity, that is, surface polarity contrast between lobes.^36^ It denotes the ability of the amphiphile to adsorb and partition at the oil–water or air/water interfaces. Different methods for quantification of amphiphilicity have been summarized in the review.^36^ By analogy to hydrophilic–liopophilic balance defined for surfactants, the proposed parameters characterize the difference in affinities of Janus particles for water and oil phases. In our case, as ε_BO_^*^ increases, the amphiphilicity of the Janus particle also increases.

Figure 4 presents examples of equilibrium configurations of the systems involving different Janus dimers. To illustrate changes in the structure of the oil aggregate, in left panels (b–f), the dimers are not shown. In the case of weak interactions between segments *B* and oil molecules (a, b), the nanoparticles adsorb at the top of the oil aggregate. The adsorbed dimers are oriented perpendicular to the substrate with the *A* parts facing outward. The oil chains lie on the surface of the substrate (b). As the parameter ε_BO_^*^ increases, more dimers tend to be in contact with oil segments, and the oil aggregate is torn and becomes looser (c). The chains are pulled away from the surface by Janus dimers (d). In the case of strong dimer-oil interactions, a kind of sandwich structure is formed, in which the oil molecules are located on the inside and the dimers are adsorbed on the outside (e). This sandwich is oriented perpendicular to the substrate and is still retained on the surface. Similar sandwich-like structures have already been observed at the oil/water interface.^28,37^ Despite Janus dimers not being attracted by the substrate, they are adsorbed at the surface due to the hydrophobicity of segments *B*. Janus dimers penetrate beneath the oil cluster and form a bridge that holds the cluster close to the substrate. These “bridging” interactions are weak, so it is easy to wash out such a cluster. Moreover, these Janus particles pull water molecules between the oil aggregate and the substrate. In this way, water channels near the solid surface are formed (Figure 4f). Notice that for stronger *BO* interactions the polymer chains are moving away from each other, and the oil clusters become looser (b, d, f). The free space is filled with diffusing water. Our simulations confirm the formation of water channels observed in previous simulations.^14,24^ The surfactant-like structure of Janus dimers strengthens the interaction between the water phase (segment *A*) and the oil aggregate (segment *B*), promoting the adsorption on the oil/water interface and detachment of the adsorbed oil cluster.

**Figure 4:**
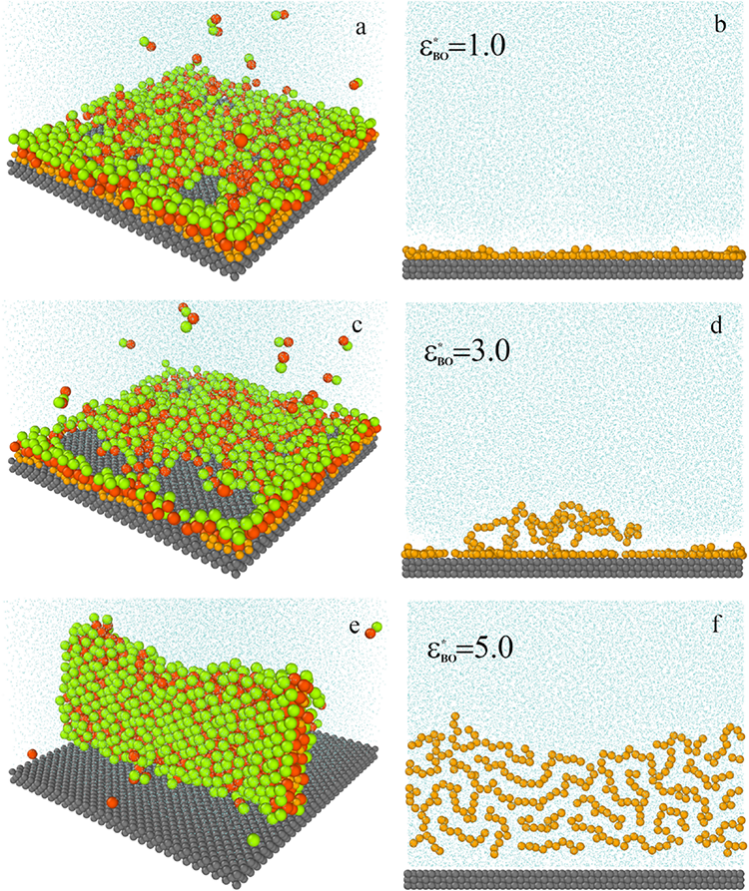
Examples of the equilibrium configurations of systems containing Janus particles with different *BO* interactions, ε_BO_^*^: 1.0 (a, b), 3.0 (c, d), and 5.0 (e, f). The green spheres represent segments *A*, the red spheres represent segments *B*, the yellow spheres correspond to the polymer segments (*O*), the light blue spheres represent the solvent (*W*), and the gray spheres represent the solid. For greater clarity of the drawing, the solvent molecules have been reduced. The density of Janus particles, ρ_AB_^*^ = 0.042.

We noticed an interesting scenario: a spherical oil droplet was observed in an aqueous solution, which was then adsorbed on the surface as a flat aggregate (Figure 1), and after adding strongly amphiphilic Janus dimers to the system (Figure 4f), it emerged from the substrate in a sandwich-like structure.

Our observations can be quantitatively confirmed by the analysis of the center of mass position of the oil droplet in the *z*-direction (z*) and the fraction of oil segments that touch the surface (*f*), calculated for increasing values of ε_BO_^*^ (Figure 5). Initially, *z*_com_^*^ and *f* remain almost constant, *z*_com_^*^ = 2.45 and *f* = 0.95. The behavior of these systems is illustrated well by snapshots in Figure 4a,b. However, for 2.5 < ε_BO_^*^ < 3.5, we observe jumps in these functions, *z*_com_^*^ quickly increases while *f* decreases. The structure of the oil aggregate changes (see Figure 4c,d). Then, both functions achieve plateaus, and *f* decreases to zero (compare Figure 4e,f). Thus, there is a certain threshold value of ε_BO_^*^ for which “kidnaping” of the oil aggregate becomes possible. Moreover, we see that an increase in amphiphilicity of Janus dimers is associated with an increase in the contact angle of the oil droplet. This tendency has been described in numerous experimental works on various Janus nanostructures, e.g., silica-based Janus nanoplatelets,^12^ SiO_2_ Janus particles combined with graphene oxide,^13^ and others.^2^

**Figure 5:**
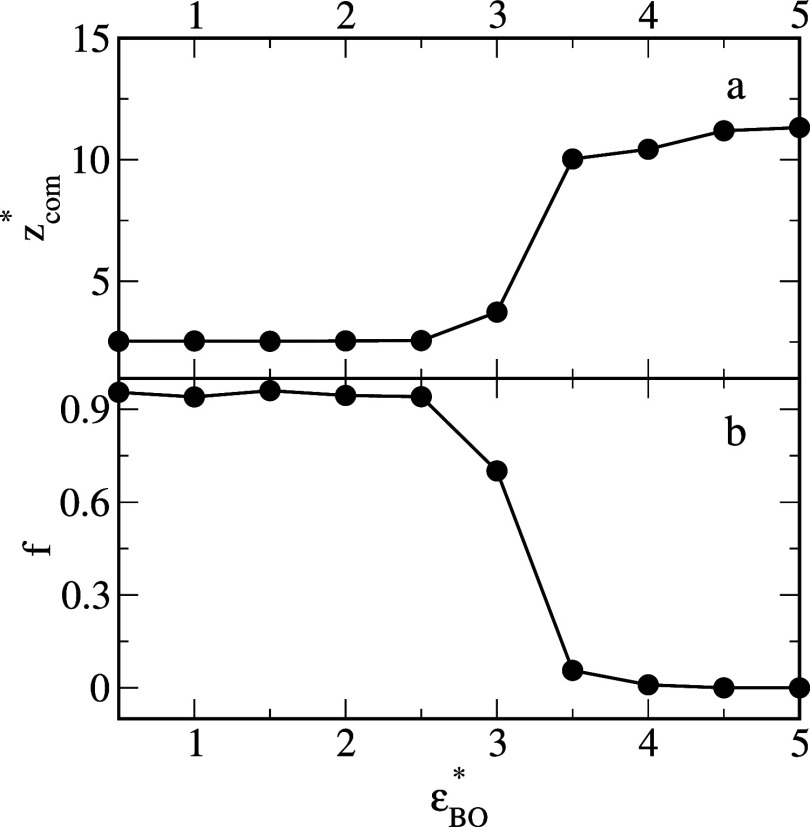
Average *z*-coordinate of the center of mass of oil segments, *z*_com_^*^, (a), and the fraction of the number of oil segments which “touch” the surface, *f*, (b) as functions of the energy parameter characterizing the *BO* interactions, ε_BO_^*^. The density of Janus particles, ρ_AB_^*^ = 0.042.

We also calculated the density profiles of oil molecules and the density profiles of segments *A* and *B* of the dimers (Figure 6). For the systems from Figure 4, ε_BO_^*^ = 1.0 (black lines), 3.0 (red lines), and 5.0 (green lines). The oil density profiles reflect well the changes in the structure of oil aggregate. For weak oil–dimer interactions, the profile has one narrow and high peak at z* = 2.53. Moreover, a low kink is visible at z* = 3.22, which indicates the formation of the second layer. As the interactions become stronger, the first peaks are somewhat lower; there is a deep minimum at z* = 3.77 and a low third peak at z* = 4.18. The wide plateau appears in the region 5.05 < z* < 9.47, and the density of oil segments sharply decreases to zero. However, for ε_BO_^*^ = 5.0, near the surface, the oil density is equal to zero and at z* = 3.77 rapidly increases to an almost constant value and falls to zero at z* = 20.34.

**Figure 6:**
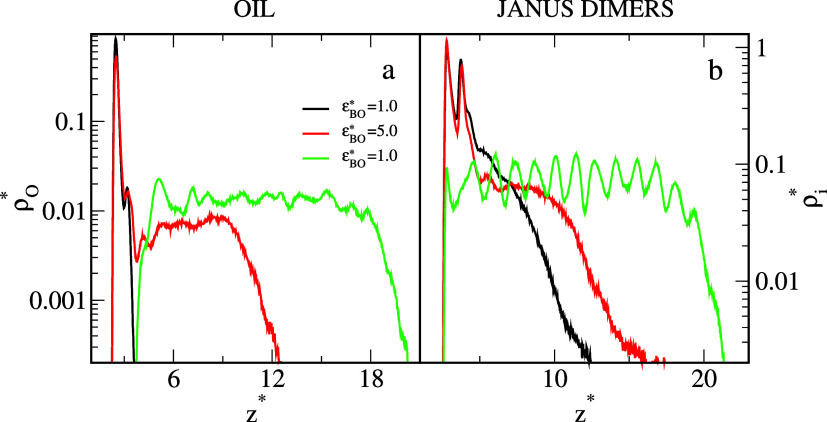
Local density profiles of the oil segments (a) and the segments of Janus dimers (b) on the strongly hydrophobic substrate for different *BO* interactions, ε_BO_^*^: 1.0 (black), 3,0 (red), and 5.0 (green). In (b), the densities of segments *B* (*A*) are plotted as solid lines (dashed lines). The abscissas are scaled logarithmically. The density of Janus particles, ρ_AB_^*^ = 0.042.

The density profiles of the dimers are correlated with the oil density profiles (Figure 6b). In the case of ε_BO_^*^ = 1.0, the dimers adsorb on the outer part of the oil aggregate. In this local density profile, ρ_B_^*^(z*), we see two peaks (at z* = 2.84 and z* = 3.79), and then the density smoothly tends to zero. The first peak corresponds to segments located around the edge of the oil stain while the second peak reflects their “adsorption” on the oil cluster. On the contrary, the density profile of segments *A*, ρ_A_^*^(z*), has three peaks near the surface. The first two peaks are associated with dimers accumulated at the edge of the oil slick and lying parallel to or tilted to the substrate. The third peak corresponds to dimers adsorbed on the droplet. Then, the densities of both segments slowly decreased to zero. The profiles of dimer segments obtained for ε_BO_^*^ = 3.0 are similar. However, the profiles calculated for ε_BO_^*^ = 5.0 have a different course. There are quite high peaks in the density of both segments close to the substrate (z* < 2.78). It indicates the presence of dimers under the oil cluster (see the oil density profile). In the region 4.24 < z* < 19.71 the segments *A* and *B* are almost evenly distributed, they are located near successive oil layers. Detailed observation indicates that segments *A* insert into the solvent, while segments *B* are strongly bonded with the oil aggregate. Such a structure of the interface could cause a significant decrease in interfacial tension.^21^ This, in turn, results in high emulsion capability, and an enhanced oil detachment.^23,32^

Comparison of Figures 6a and 2a (black line) proves that the addition of Janus particles completely changes the shape and structure of the oil aggregates anchored to the surface under equilibrium conditions.

We also explored the dynamics of the changes in the structure of the oil aggregate adsorbed on the solid surface. In Figure 7, we show the time evolution of the average *z*-coordinate of the center of mass (top panel) and the number of oil segments on the surface (bottom panel) in the presence of different Janus dimers. For weak *BO* interactions (black line), these parameters are almost constant. This means that the structure of the oil aggregate remained the same. In the case of moderate *BO* interactions (red line), we see slow changes in the observables *z*_com_^*^ and *f* until they reach the equilibrium values. However, for the strong interactions (green line), the structure of the aggregate changes quickly.

**Figure 7:**
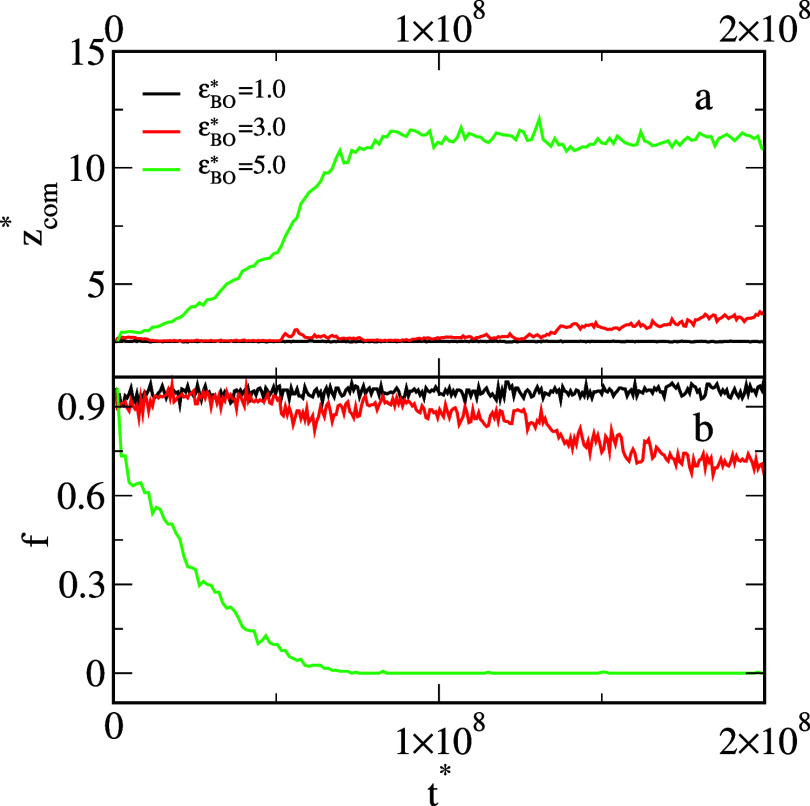
Average *z*-coordinate of the center of mass of the oil segments (a) and the fraction of the number of oil segments on the surface (b) as functions of time for different *BO* interactions, ε_BO_^*^: 1.0 (black), 3.0 (red), and 5.0 (green). The density of Janus particles, ρ_AB_^*^ = 0.042.

Let us now move on to the analysis of the influence of Janus dimer concentration on the behavior of oil aggregates on the surface. These simulations were performed for strongest *BO* interactions (ε_BO_^*^ = 5) and different densities of dimers. The remaining energy parameters were the same as those above.

In Figure 8, we show examples of equilibrium configurations for systems with increasing density of dimers, from ρ_AB_^*^ = 0 to ρ_AB_^*^ = 0.035. We see that dimers gradually cover the surface of the oil aggregate which causes its reconfiguration. The shape of the aggregate changes from a flat “puddle” lying on the substrate to a plate in contact with the surface on its narrower side. Further increase in ρ_AB_^*^ leads to the structure shown in Figure 4f. The internal structure of the aggregate also changes, the chains are moving away from each other, and the oil cluster becomes less compact (not shown here).

**Figure 8:**
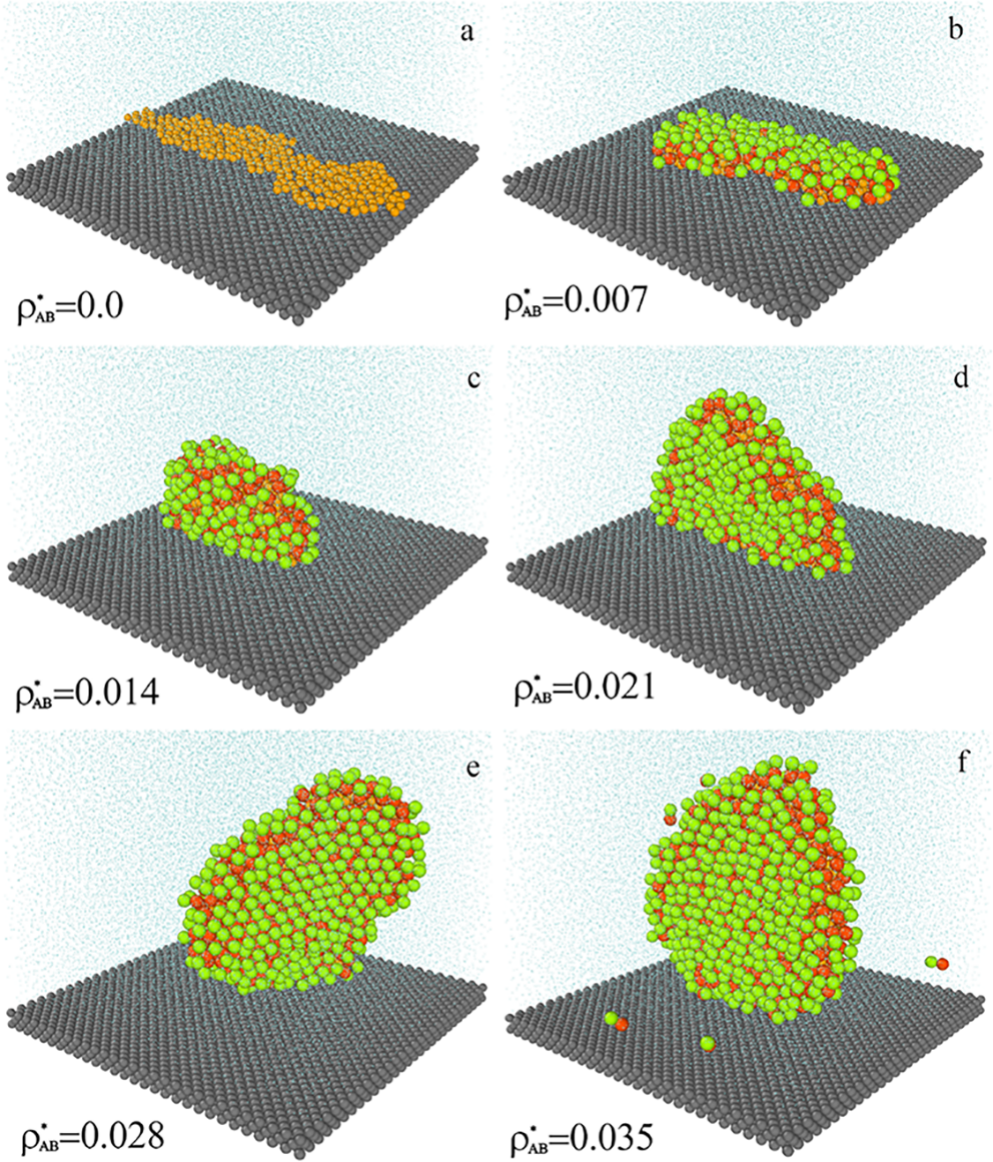
Examples of the equilibrium configurations of systems with different densities of Janus particles ρ_AB_^*^: 0 (a), 0.007 (b), 0.014 (c), 0.021 (d), 0.028 (e), and 0.035 (f). The green spheres represent segments *A*, the red spheres represent segments *B*, the yellow spheres correspond to the polymer segments (*O*), the light blue spheres represent the solvent (*W*), and the gray spheres are for the solid. For greater clarity of the drawing, the solvent molecules have been reduced. The parameter ε_BO_^*^ = 5.0.

Figure 9 shows the density profiles for different average densities of the dimers. When ρ_AB_^*^ = 0.007 (black line), the oil density profile has three narrow peaks near the surface and sharply decreases to zero at z* = 5.01 (a). As the average dimer density increases slightly (red and green lines), these peaks become lower; then, the density reaches an almost constant value and rapidly decreases to zero. For sufficiently high density ρ_AB_^*^ (blue and yellow lines), the oil density near the surface equals zero, for z* > 4.95, the oil density jumps to a plateau and then falls to zero. The density profiles of segments *A* and *B* (b) confirm the adsorption of dimers on the oil aggregate. For high densities, ρ_AB_^*^ dimers squeeze between the oil aggregate and the substrate, which leads to a gradual detachment of the oil cluster. The profiles of segments *A* are shifted toward the bulk phase. A higher concentration of Janus dimers led to their accumulation in the three-phase contact area could also exert a structural separation pressure, causing trapped oil to be separated from the surface.^23^

**Figure 9:**
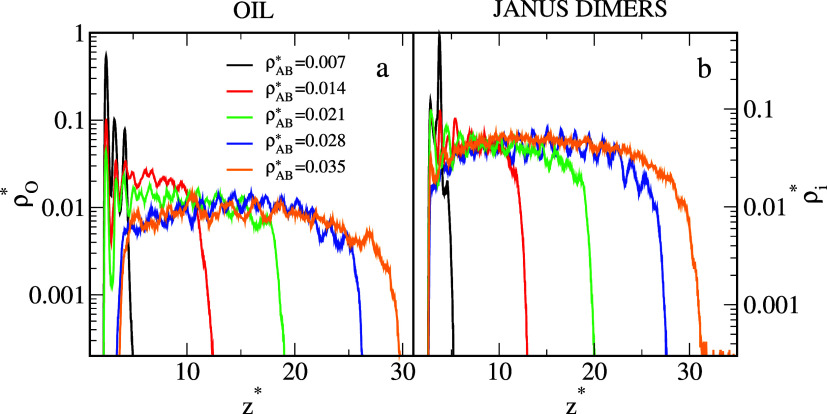
Local density profiles of the oil segments (a) and the segments of Janus dimers (b) on the strongly hydrophobic substrate for different average densities of Janus dimers, ρ_AB_^*^: 0.007 (black), 0.014 (red), 0.021 (green), 0.028 (blue), and 0.035 (orange). In (b), the densities of segments *B* (*A*) are plotted as solid lines (dashed lines). The abscissas are scaled logarithmically. The parameter ε_BO_^*^ = 5.0.

In Figure 10, *z*_com_^*^ and *f* are plotted as functions of the average dimer density. An increase in the density of Janus dimers causes a gradual increase in *z*_com_^*^ and a decrease in *f*. This is consistent with the experimental observations that showed that at a higher concentration of Janus particles, the contact angle of the oil droplet was larger.^13^ Moreover, when more Janus dimers were added to the system, the reconfiguration of oil aggregate was much quicker (see Figure 11).

**Figure 10:**
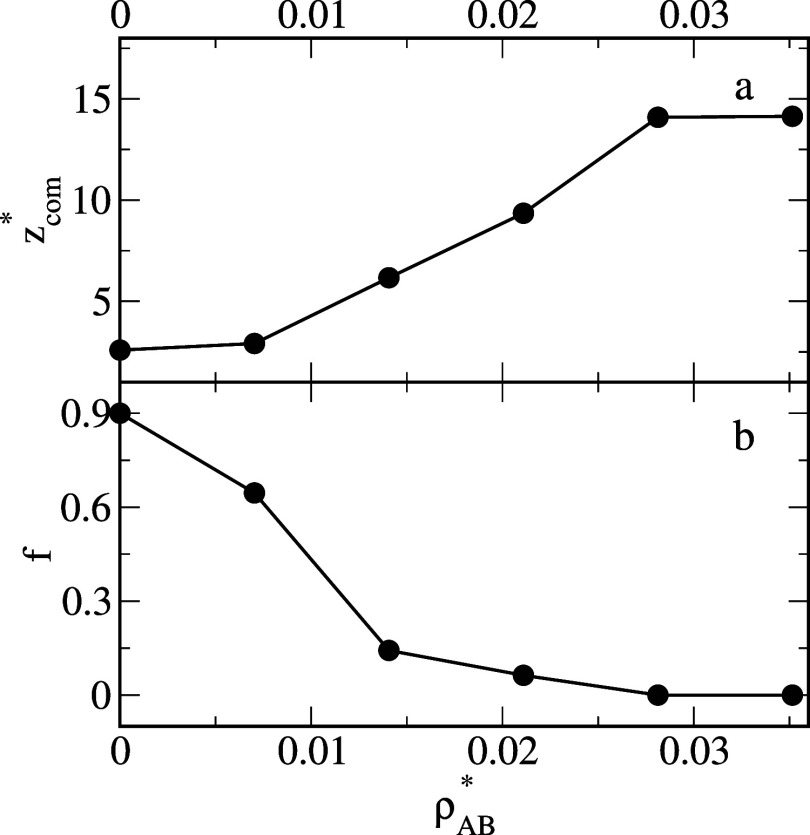
Average *z*-coordinate of the center of mass of oil segments, *z*_com_^*^, (a) and the fraction of the number of oil segments which “touch” the surface, *f*, (b) as functions of the density of Janus dimers ρ_AB_^*^. The parameter ε_BO_^*^ = 5.0.

**Figure 11:**
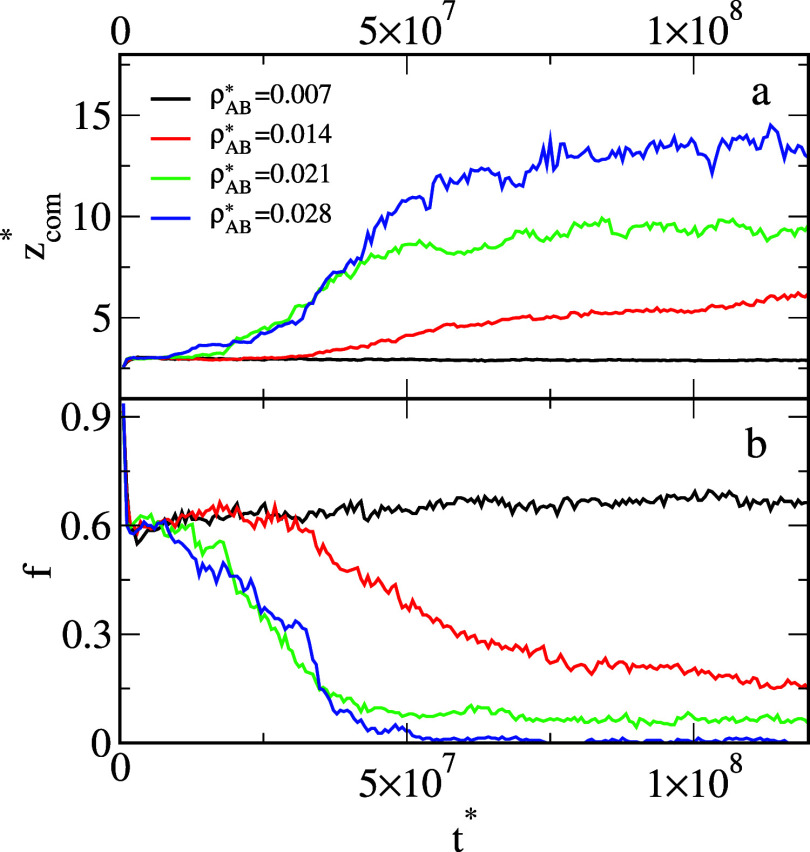
Average *z*-coordinate of the center of mass of the oil segments, *z*_com_^*^, (a) and the fraction of the number of oil segments on the surface, *f*, (b) as functions time for different densities of Janus dimers, ρ_AB_^*^: 0.007 (black), 0.014 (red), 0.021 (green), and 0.028 (blue). The parameter ε_BO_^*^ = 5.0.

To sum up, interactions of Janus nanoparticles with oil aggregates lead to changes in their shape and internal structure and can cause their detachment from the substrate. One can say that Janus particles take up the oil aggregate from the surface of the solid. This process can be controlled by either the amphiphilicity of Janus dimers or the average concentration of Janus dimers. The mechanism of oil detachment described here was suggested in experimental work concerning Janus SiO_2_ particles amalgamated with graphene oxide.^13^ The effects of amphiphilicity and concentration of Janus particles on the separation of oil from solid surfaces observed here are consistent with the results of previous simulations^15,20,21,31,35^ and experimental works.^12,13,38^

The simulations discussed above have shown that Janus dimers with sufficiently high amphiphilicity facilitate oil detachment from the substrate. Currently, the methods are known to synthesize the Janus dimers with gradually increased amphiphilicity.^36^ By performing a series of measurements for different Janus dimers, it would be possible to determine the limiting value of amphiphilicity, from which a drop of oil spontaneously detaches from the surface. Similarly, one can determine the optimal concentration of the Janus particles. The results provided guidance for designing suitable Janus dimers and choosing their concentration.

### 3.3 Adsorption of Janus Particles on the Substrate

We also performed simulations for Janus particles that were attracted by the substrate. Our goal was to analyze the role of Janus particle-substrate interactions in oil removal. For this purpose, we assumed that BS interactions were attractive (ε_BS_^*^ = 5) while segments *A* were inert to the substrate. Moreover, both segments of Janus dimers were assumed to be inert to the oil.

We introduced to the system *N*_*O*_ = 800 Janus dimers (ρ_AB_^*^ = 0.056) and observed its time evolution. In Figure 12, we see that dimers slowly accumulate at the substrate, replacing oil particles on the surface. The mechanism of this process is very interesting. Initially, a large flat oil slick (a) is torn into smaller, also flat ones (b, c). Then, the oil clusters change their shapes. They form droplets that gradually elongate in the direction normal to the substrate (d). These droplets detach from the surface, one by one (e). In the bulk phase, they merge back into one drop (e).

**Figure 12:**
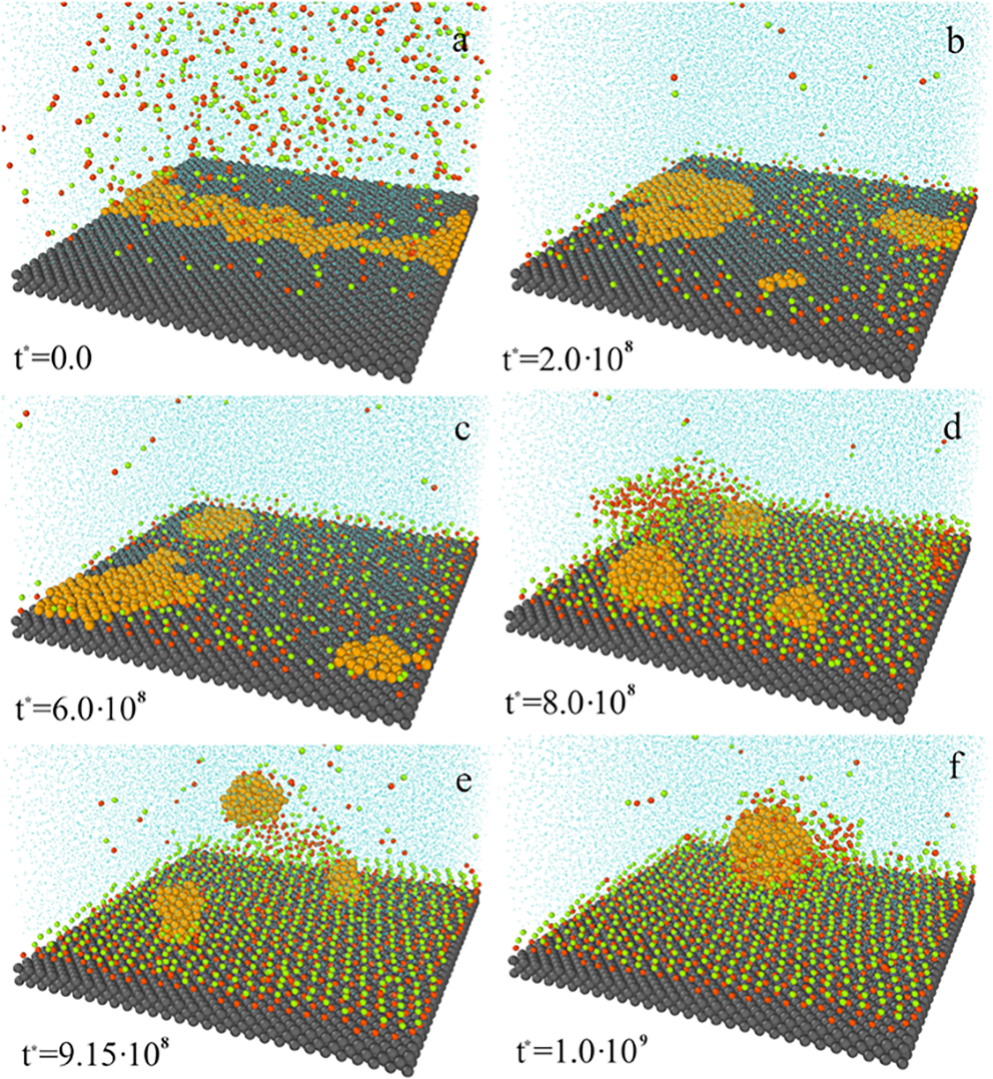
Time evolution of the system involving Janus dimers that are attracted by the substrate, ε_AS_^*^ = 5.0. The simulation time: 0 (a), 2.0·10^8^ (b), 6.0·10^8^ (c), 8.0·10^8^ (d), 9.15·10^8^ (e), and 1.0·10^9^ (f). The green spheres represent segments *A*, the red spheres represent segments *B*, the yellow spheres correspond to the polymer segments (*O*), the light blue spheres represent the solvent (*W*), and the gray spheres are for the solid. For greater clarity of the drawing, the solvent molecules and dimer segments have been reduced. Janus dimers are inert with respect to the oil and solvent. The density of Janus particles, ρ_AB_^*^ = 0.056.

In Figure 13, the final stage of this process is shown in more detail. We start with three small droplets attached to substrate (a). Then, one of them (the middle droplet) breaks away from the surface (b), and it sticks to the droplet still attached to the surface (the one on the right) (c). This large droplet leaves the surface (d). The situation repeats itself; the free drop merges with the last drop bound to the surface, and the whole oil cluster leaves the substrate.

**Figure 13:**
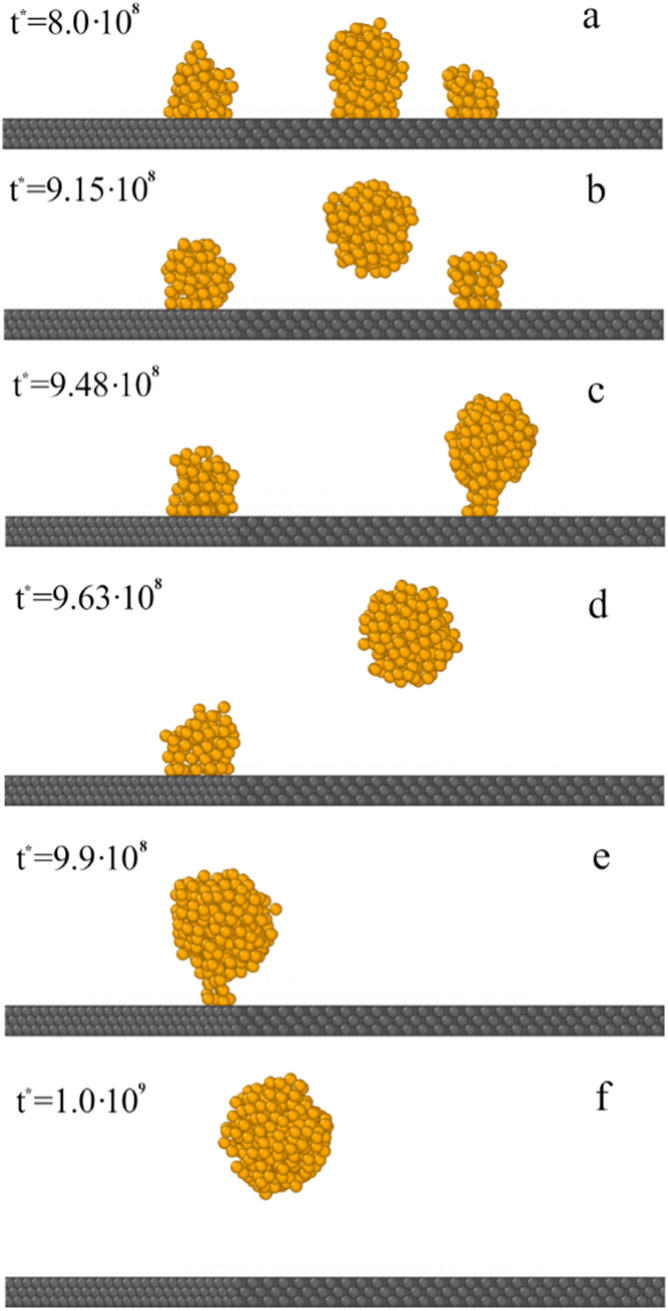
Final stage of oil droplet detachment is shown in Figure 12. The simulation time: 8·10^8^ (a), 9.15·10^8^ (b), 9.48·10^8^ (c), 9.63·10^8^ (d), 9.90·10^8^ (e), and 1.00·10^9^ (f). The yellow spheres correspond to polymer segments (*O*). For greater clarity of the drawing, the solvent molecules and Janus dimers have been omitted.

The function *z*_com_^*^(t*) reflects the subsequent stages of oil removal very well (Figure 14a). We see here three peaks and two minima marked by red arrows with letters corresponding to snapshots shown in Figure 13. The subsequent attachments and detachments of the droplets are clearly visible. However, the number of oil segments on the surface smoothly decreases as time passes (Figure 14b).

**Figure 14:**
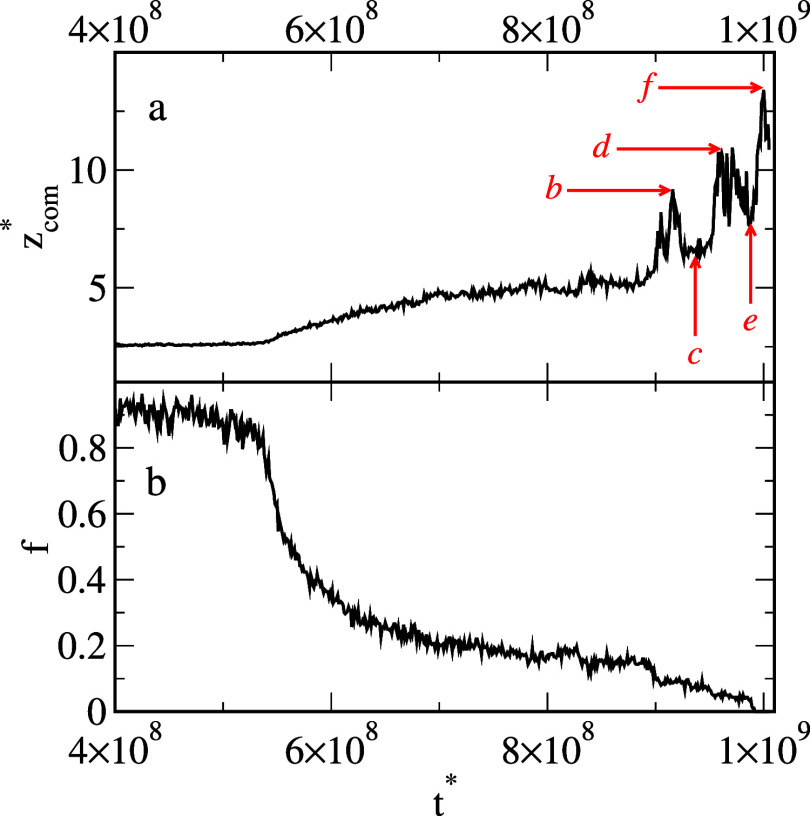
Average *z*-coordinate of the center of mass of the oil segments, *z*_com_^*^, (a) and the number of oil segments on the surface, *f*, (b) as functions of time for the system from Figure 13. The red arrows have letters corresponding to the snapshots shown in Figure 13.

Our results are consistent with the simulations performed by Chang et al.^26^ for spherical Janus nanoparticles with different ratios of polar and nonpolar parts on the surfaces. They showed that the competitive adsorption of Janus nanoparticles could be the first step in oil recovery from rough nanochannels. It was shown that Janus particles with half a nonpolar surface adsorbed on the flat surface and at the oil–water interface but did not penetrate the oil phase. We observed the same behavior for Janus dimers (see Figure 12). It should be stressed, however, that we considered the static system at equilibrium, while Chang’s group dealt with the flooding nanofluids. Moreover, they did not consider the effects of the concentration of Janus nanoparticles.

The simulations discussed here have shown that the competitive adsorption of Janus particles on the substrate can play a significant role in oil recovery. However, the displacement of oil molecules by Janus particles on the surface requires the application of high concentrations of Janus particles.

In real systems, the two mechanisms described above occur simultaneously. Janus particles can either draw oil particles into the bulk phase or displace them from the surface. Simulations allowed us to explore these processes separately.

## 4 Conclusions

In summary, by employing coarse-grained molecular dynamics simulations, we investigated the deposition of oil droplets on the different substrates and their detachment induced by Janus particles were investigated. The selected parameters were proven to successfully reproduce the nature of the components in the systems considered. To test our coarse-grained approach, we analyzed how solvent (water)-surface interactions influence the structure of the adsorbed oil clusters. We proved that the designed model predicted well the shape transformation of oil droplets adsorbed on different surfaces. On the strongly hydrophobic surfaces, the oil droplet adsorbs as a flat compact cluster. However, on hydrophilic substrates, the oil droplet does not adsorb at all.

Then we focused on the behavior of the oil clusters attached to the strongly hydrophobic surfaces. Removing oil from such surfaces is extremely difficult. We studied the detachment of the oil clusters from the surface induced by Janus dimers. We consider two mechanisms of the process: (i) the detachment of the oil cluster from the surface by Janus dimers and (ii) the displacement of the oil molecules by Janus dimers on the substrate through competitive adsorption.

The “kidnaping” contains two stages: the desorption of oil from the substrate and the formation of dispersed oil droplets (emulsification) in the solvent. To simplify the analysis, we assumed that Janus dimers were inert with respect to the surface. Such a “kidnaping” can be controlled either by the amphiphilicity of Janus particles or by the average concentration of Janus dimers. We found that

(i) Adsorption of Janus dimers on the oil aggregate changed its shape and internal structure. For sufficiently high amphiphilicity of Janus dimers, the unique flat sandwich-like structure was formed.
(ii) There was a threshold value of amphiphilicity at which Janus dimers could draw a droplet into the aqueous phase, while an increase in the concentration of Janus dimers gradually enhanced the oil detachment.
(iii) The detachment of the oil cluster from the substrate was faster when the interactions of the Janus particles with the oil molecules were stronger or as the average density of Janus dimers became higher.
(iv) In the “kidnaping” process, the flat oil aggregate left the surface as a large, sandwich-like aggregate.

The observed trends are consistent with those reported in previous simulations^15,20,21,31,35^ and experiments.^2,5,12,22,38^

The mechanism of the displacement of oil molecules on the surface by Janus dimers through competitive adsorption was also analyzed in detail. No systematic studies of this effect have been conducted so far. We found that the detachment of the oil cluster takes place in several stages, including breaking a flat stain into smaller ones, the elongation of oil clusters in the direction normal to the substrate, the detachment of elongated clusters, and the detachment of another droplet from the surface by the free droplets.

The current simulation results may help to understand the interaction between the Janus nanoparticles and oil droplets attached to the hydrophobic substrate and give some new insights into the possible mechanism of the EOR process. The study provides insight into the detachment of oil aggregates strongly adsorbed on the solid surface, which has hardly been investigated by the relevant experiments. This study paves an avenue for the planning of suitable experiments and future studies on the mechanism of the nanoparticle-induced detachment of oil from other substrates at the nanoscale. Furthermore, our findings can be adopted in the design and screening of Janus particles for enhanced oil recovery. In particular, by performing a series of measurements for different Janus dimers, it would be possible to determine the limiting value of amphiphilicity of Janus dimers from which a drop of oil spontaneously detaches from the surface and to determine their optimal concentration. The results guided the application of amphiphilic nanoparticles in enhanced oil recovery.
